# Serum 8-OHdG as a Marker of Oxidative DNA Damage in Acute Exacerbations of COPD: Associations with GOLD Stage and Smoking Status—A Cross Sectional Study

**DOI:** 10.3390/medicina62071369

**Published:** 2026-07-16

**Authors:** Larisa Alexandra Rus, Romana Olivia Popețiu, Simona Maria Borta, Anamaria Vîlcea, Nicolae Cătălin Hreniuc, Adrian Silviu Crișan, Carla Melania Tamaș, Paula Alexandra Vulciu, Oana Știrbu, Cecilia Avram, Denisa Goldiș, Darius Radu Roman, Alexandru Chioreanu, Radmila-Anca Bugari, Dana Zdremțan, Cristina Georgiana Firu, Imola Donath-Miklos, Luminița Pilat, Maria Pușchiță

**Affiliations:** 1 Department of Internal Medicine, Faculty of Medicine, “Vasile Goldiș” Western University of Arad, B-dul Revoluției nr. 94–96, 310025 Arad, Romania; larisa_gal@yahoo.com (L.A.R.); popetiur@gmail.com (R.O.P.); anamariavilcea33@gmail.com (A.V.); oanastirbu66@yahoo.com (O.Ș.); mpuschita.mp@gmail.com (M.P.); 2 Arad County Emergency Clinical Hospital, Str. Andrényi Károly Nr. 2–4, 310037 Arad, Romania; cata_hr@yahoo.com (N.C.H.); adriancrisan74@yahoo.com (A.S.C.); carla_tamas@yahoo.com (C.M.T.); avramcici02@yahoo.com (C.A.); timisdenisa333@yahoo.com (D.G.); 3 Department of Neurology, Faculty of Medicine, “Vasile Goldiș” Western University of Arad, B-dul Revoluției nr. 94–96, 310025 Arad, Romania; 4 Department of Critical Care and Emergency Medicine, Faculty of Medicine, “Vasile Goldiș” Western University of Arad, B-dul Revoluției nr. 94–96, 310025 Arad, Romania; 5 Department of Biochemistry, Faculty of Medicine, “Vasile Goldiș” Western University of Arad, B-dul Revoluției nr. 94–96, 310025 Arad, Romania; vulciu.paula@uvvg.ro (P.A.V.); zdremtan.dana@uvvg.ro (D.Z.); luminita.pilat@yahoo.com (L.P.); 6 Department of Biology and Life Science, Faculty of Medicine, “Vasile Goldiș” Western University of Arad, B-dul Revoluției nr. 94–96, 310025 Arad, Romania; 7 Clinical Laboratory of Medical Analyses, Arad County Emergency Clinical Hospital, Str. Andrényi Károly nr 2–4, 310037 Arad, Romania; 8 Doctoral School of Biomedical Sciences, Faculty of Medicine, and Pharmacy, University of Oradea, Str. Universității nr 1, 410087 Oradea, Romania; roman.dariusradu@student.uoradea.ro; 9 Department of Otorinolaryngology, Faculty of Medicine, “Vasile Goldiș” Western University of Arad, B-dul Revoluției nr. 94–96, 310025 Arad, Romania; chioreanu.alexandru@uvvg.ro (A.C.); radmilabugariturcin@gmail.com (R.-A.B.); 10 Department of Heamatology, Faculty of Medicine, “Vasile Goldiș” Western University of Arad, B-dul Revoluției nr. 94–96, 310025 Arad, Romania; paracris17@yahoo.com

**Keywords:** chronic obstructive pulmonary disease, acute exacerbation, 8-hydroxy-2′-deoxyguanosine, oxidative stress, biomarkers, smoking, airflow limitation, disease severity, GOLD classification, systemic inflammation

## Abstract

*Background and Objectives:* Oxidative stress is widely recognized as a key contributor to both the development and progression of chronic obstructive pulmonary disease (COPD), particularly during acute exacerbations (AECOPD). 8-Hydroxy-2′-deoxyguanosine (8-OHdG), a marker of oxidative DNA damage, has been insufficiently investigated as a systemic biomarker in this setting. This study evaluated the relationship between serum 8-OHdG levels, airflow limitation severity, and smoking status in patients hospitalized with AECOPD. *Materials and Methods:* We conducted a cross-sectional study including 176 patients admitted for AECOPD and stratified according to Global Initiative for Chronic Obstructive Lung Disease (GOLD) stages. The study population comprised patients with comparable distributions of age, sex, smoking status, diabetes, cardiovascular comorbidities, and area of residence across GOLD categories. Serum 8-OHdG, leukocyte count, neutrophil percentage, C-reactive protein (CRP), fibrinogen, and procalcitonin were measured. Group comparisons, multivariable logistic regression analyses, and smoking-status subgroup analyses were performed. *Results:* Serum 8-OHdG levels increased significantly with advancing airflow limitation severity (*p* = 0.038), with higher concentrations observed in patients with GOLD 4 disease compared to GOLD 1–2 disease (*p* = 0.023). In multivariable analysis, 8-OHdG was the only biomarker independently associated with GOLD stage (OR = 2.44, 95% CI: 1.12–5.31, *p* = 0.025). Higher serum 8-OHdG levels were also independently associated with smoking status (OR = 1.20, 95% CI: 1.05–1.37, *p* = 0.022). Ever-smokers demonstrated significantly higher 8-OHdG concentrations than never-smokers in GOLD 1–2 and GOLD 3 disease, whereas procalcitonin levels were higher among never-smokers with advanced COPD. *Conclusions:* Serum 8-OHdG levels measured during AECOPD are associated with both airflow limitation severity and smoking status. These findings support the potential role of oxidative DNA damage as a clinically relevant component of COPD pathophysiology and suggest that serum 8-OHdG may represent a useful biomarker for disease characterization in patients experiencing acute exacerbations.

## 1. Introduction

Chronic obstructive pulmonary disease (COPD) is a highly prevalent respiratory disorder associated with substantial morbidity and mortality worldwide. Persistent airflow limitation, chronic inflammation, and excessive oxidative stress represent the principal pathological hallmarks of the disease [1,2,3,4,5]. Associated with adverse outcomes, increased clinical burden, and heterogeneous symptomatology, acute exacerbations (AECOPDs) are critical events in COPD progression [4,6,7,8,9]. The identification of biomarkers reflecting the pathophysiological dynamics of these events is therefore an area of active investigation [8,9,10,11,12,13]. Markers of oxidative stress and inflammation are particularly relevant in this context since both these processes are intertwined drivers of COPD pathogenesis [8,12].

Because it reflects cumulative oxidative DNA damage, 8-hydroxy-2′-deoxyguanosine (8-OHdG) has attracted interest as a potential biomarker of disease activity and progression in chronic inflammatory disorders.

This modified DNA nucleoside results from hydroxyl radical attack on guanine bases and its expression is linked to the severity of inflammation [14]. The measured levels in tissues, blood, or urine increase following exposure to various environmental factors, including air pollutants and volatile organic compounds [15,16]. This parameter is also sensitive to oxidative stress caused by smoking—the most important driver of COPD [17]. In fact, clinical evidence revealed an increased total amount of 8-OHdG in the lungs of smokers versus non-smokers [13], with heightened concentrations seen in ex-smokers even post-cessation and further elevated levels in smokers who develop this disease [13]. However, there is surprisingly little information about its systemic levels in COPD. Evidence from blood-derived fluids is scarce, as only a limited number of clinical studies have evaluated circulating 8-OHdG levels in COPD patients [18,19,20,21]. In addition, the dynamics of 8-OHdG across the spectrum of airflow limitation—a fundamental hallmark of COPD [4]—are still far from being fully understood. Moreover, the interplay between smoking status, oxidative stress, and airflow obstruction remains an open question [18].

Conventional inflammatory biomarkers, including leukocyte count, neutrophil percentage, CRP, fibrinogen, and procalcitonin, are frequently used during AECOPD assessment [8,10,11,22,23,24,25]. These markers provide information regarding systemic inflammation and infectious triggers, but their relationship with oxidative DNA damage remains incompletely understood [26,27,28,29,30,31,32,33].

The present study aimed to evaluate the relationship between serum 8-OHdG levels, airflow limitation severity, and smoking status in patients hospitalized with AECOPD. In addition, we compared the performance of 8-OHdG with commonly used inflammatory biomarkers, including leukocyte count, neutrophil percentage, CRP, fibrinogen, and procalcitonin. We hypothesized that serum 8-OHdG would show a stronger association with airflow limitation severity and smoking status than conventional inflammatory markers.

## 2. Materials and Methods

### 2.1. Study Design

This exploratory cross-sectional study was carried out between May 2024 and April 2025 at the Arad County Emergency Clinical Hospital (SCJU Arad), Romania. The hospital serves a population of more than 400,000 inhabitants and includes an 83-bed Department of Pneumology [34], providing an appropriate clinical setting for the present investigation. The study protocol received approval from the independent ethics committees of both SCJU Arad (Approval No. 38/15 May 2024) and “Vasile Goldiș” Western University of Arad (Approval No. 17/26 March 2024). Written informed consent was obtained from all participants or, when applicable, from their legal representatives before enrollment.

An acute exacerbation of chronic obstructive pulmonary disease (AECOPD) was defined as an acute deterioration in the patient’s baseline respiratory condition, characterized by worsening dyspnea, persistent cough, increased sputum production and/or sputum purulence, requiring treatment beyond the regular maintenance regimen [4].

Eligible participants met the following inclusion criteria: (i) age ≥ 40 years; (ii) a confirmed diagnosis of COPD according to GOLD criteria, defined by a post-bronchodilator forced expiratory volume in one second (FEV_1_)/forced vital capacity (FVC) ratio < 0.70; (iii) hospitalization for AECOPD; (iv) documented smoking status; and (v) complete availability of the required clinical and laboratory data.

The common exclusion criteria were as follows: (i) other chronic pulmonary diseases (e.g., asthma, bronchiectasis, pulmonary fibrosis); (ii) respiratory infection unrelated to AECOPD within the last four weeks; (iii) administration of systemic corticosteroids for more than 72 h prior to admission; (iv) active malignancy (or known immunosuppressive condition); (v) chronic renal or liver failure; (vi) pregnancy or breastfeeding; and (vii) inability to perform spirometry or comply with study procedures. None of the patients received additional treatment (intervention) for the current exacerbation episode before admission to the hospital.

At baseline, 293 AECOPD patients were recruited and stratified into three groups of airflow limitation severity, i.e., mild-to-moderate (GOLD 1–2), severe (GOLD 3), and very severe (GOLD 4). Individuals with GOLD stages 1 and 2 were consolidated into a single analytical category given their similarities in terms of lung function, symptom severity, and exacerbation risk [34,35]. Patients were selected to achieve comparable distributions of age, sex, area of residence, smoking status, diabetic status, and cardiovascular comorbidities across GOLD categories. These variables represent well-recognized confounding factors in COPD and are therefore important to consider when investigating biomarker profiles and clinical outcomes [3,34,35,36,37]. Following application of the eligibility criteria, a total of 176 patients were included in the final analysis.

Patients were recorded as living in either rural or urban areas according to the official residence recorded in their medical records. Smoking status was categorized as ever smoker (including both current and former smokers) or never smoker. Consistent with standard epidemiological definitions, an ever smoker was defined as an individual who had smoked at least 100 cigarettes during their lifetime, regardless of current smoking behavior [38]. Diabetes mellitus was diagnosed based on established criteria, including a fasting plasma glucose concentration of ≥7.0 mmol/L (≥125 mg/dL), a glycated hemoglobin (HbA1c) level of ≥6.5% (48 mmol/mol), or a random plasma glucose level > 200 mg/dL in the presence of typical hyperglycemic symptoms [39]. Cardiovascular comorbidity comprised disorders affecting the heart and vascular system, including coronary artery disease, cerebrovascular disease, peripheral arterial disease, heart failure, cardiac arrhythmias, hypertensive heart disease, and valvular heart disease [40].

### 2.2. Measurements

Peripheral blood specimens were obtained during the first 24 h following hospital admission, using standard aseptic techniques to reduce preanalytical variability and contamination risk. Laboratory analyses were performed as soon as possible after venipuncture. Absolute leukocyte count and neutrophil percentage were assessed using the SYSMEX XN-1500 hematology analyzer (Sysmex Corporation, Kobe, Japan) [23]. CRP values were measured by immunoturbidimetry (Cobas analyzer; F. Hoffmann-La Roche Ltd., Basel, Switzerland). Plasma fibrinogen concentrations were quantified using the Clauss method and the STA Compact Max 3 analyzer (Diagnostica Stago S.A.S., Asnières sur Seine, France). Procalcitonin levels were assessed by quantitative electrochemiluminescence using the COBAS e 601 module (on the same platform). Serum 8-OHdG was measured with a competitive ELISA kit (code UNES00018; Assay Genie, Dublin, Ireland). This kit offers a detection range of 1.56–100 nanograms per milliliter (ng/mL), with a sensitivity of 0.94 ng/mL [41]. All assays were performed in triplicate to ensure reproducibility and minimize intra-assay variability [42].

Spirometry, by contrast, was performed during the clinically stable period [4], typically 0.5–2.5 months following recovery from AECOPDs. This time frame is consistent with the overall clinical consensus on the optimal time to reassess GOLD stage post-exacerbation [43,44].

### 2.3. Statistical Analysis

Chi-square (χ^2^) tests were applied to compare the distributions of categorical variables across GOLD stages [45]. Sex (female = 0, male = 1), origin area (rural = 0, urban = 1), smoking status (never smoker = 0, ever smoker = 1), diabetic status (no = 0, yes = 1), and cardiovascular comorbidity (no = 0, yes = 1) were coded as binary variables. Kruskal–Wallis tests were used to analyze differences in continuous variables, including age and all biological metrics analyzed. In case of significant differences, post hoc pairwise comparisons against GOLD 1–2 (mild-to-moderate COPD) were conducted using Dunn’s tests with Bonferroni correction. The corresponding effect sizes were estimated using epsilon squared (ε^2^) and interpreted as small (ε^2^ ≈ 0.01), moderate (ε^2^ ≈ 0.06), or large (ε^2^ ≥ 0.14) [46].

Before fitting logistic regression models, variance inflation factors (VIFs) were calculated for all biological variables analyzed to detect multicollinearity. A threshold of VIF < 5 was used to exclude problematic multicollinearity [47]. Ordinal logistic regression was applied on laboratory parameters to identify the best predictors of airflow limitation severity during exacerbation events. A binary logistic regression was then used to determine the biological metrics associated with smoking in AECOPD individuals. For significant predictors, biomarker levels in ever smokers and never smokers were compared using Mann–Whitney U tests. All data were processed with the Statistica 10 software package (StatSoft Inc, Tulsa, OK, USA). A two-tailed *p* value less than 0.05 was considered significant [48].

### 2.4. Artificial Intelligence Disclosure

In preparing this manuscript, the authors used OpenAI ChatGPT (GPT-5 series) solely to support language refinement, structural organization, and the development of figure concepts and graphical layouts. The tool was not involved in data acquisition, statistical analysis, result interpretation, or the formulation of scientific conclusions. All AI-assisted content was independently checked, revised, and approved by the authors, who retain full responsibility for the accuracy and integrity of the manuscript.

## 3. Results

### 3.1. Baseline Clinical and Laboratory Data

Data related to provenience area, sex, smoker/non-smoker status, glycemic balance, and cardiovascular diseases are given in Table 1. Frequency analysis showed no significant differences across GOLD stages with respect to area of residence (χ^2^ test, *p* = 0.218), sex (χ^2^ test, *p* = 0.702), smoking status (χ^2^ test, *p* = 0.910), diabetic status (χ^2^ test, *p* = 0.192), or cardiovascular comorbidity (χ^2^ test, *p* = 0.429).

The median values and interquartile ranges of continuous variables are presented in Table 2. Serum CRP and fibrinogen concentrations were generally above the reference range, whereas the remaining biomarkers were largely within normal limits. Among the laboratory parameters analyzed, serum 8-OHdG showed a significant increase across GOLD stages, while no significant differences were observed for age, leukocyte count, neutrophil percentage, CRP, fibrinogen, or procalcitonin.

Age was similar across GOLD stages. Among biomarkers analyzed, only serum 8-OHdG revealed significant interstrata differences—with an associated ε^2^ indicating a moderate effect (Table 2).

Age did not differ significantly across GOLD stages. Among the biomarkers analyzed, only serum 8-OHdG demonstrated significant intergroup differences (*p* = 0.038), with an associated moderate effect size (ε^2^ = 0.12). Post hoc analysis showed significantly lower serum 8-OHdG levels in patients with mild-to-moderate COPD (GOLD 1–2) compared with those with very severe disease (GOLD 4; Dunn’s test, *p* = 0.023). No significant differences were observed between GOLD 1–2 and GOLD 3 patients (Dunn’s test, *p* = 0.476).

### 3.2. Predictors of GOLD Stage During AECOPDs

The results of the ordinal logistic regression analysis are summarized in Table 3. All predictors showed low multicollinearity (VIF < 2). Among the biomarkers analyzed, serum 8-OHdG was the only variable independently associated with airflow limitation severity. Each unit increase in serum 8-OHdG was associated with a 2.44-fold increase in the odds of belonging to a more severe GOLD category (OR = 2.44, 95% CI: 1.12–5.31, *p* = 0.025). No significant associations were observed for leukocyte count, neutrophil percentage, CRP, fibrinogen, or procalcitonin.

### 3.3. Predictors of Smoking Status

The results of the binary logistic regression analysis are presented in Table 4. All predictors showed low multicollinearity (VIF < 2). Among the biomarkers analyzed, serum 8-OHdG was the only variable independently associated with smoking status. Each unit increase in serum 8-OHdG was associated with a 20% increase in the odds of being an ever smoker (OR = 1.20, 95% CI: 1.05–1.37, *p* = 0.022). No significant associations were observed for leukocyte count, neutrophil percentage, CRP, fibrinogen, or procalcitonin.

The median biomarker values according to smoking status and GOLD stage are presented in Table 5. Ever smokers with mild-to-moderate COPD (GOLD 1–2) showed significantly higher serum 8-OHdG levels than never smokers (*p* = 0.045). Similar findings were observed in patients with severe COPD (GOLD 3; *p* = 0.042). In contrast, no significant difference in serum 8-OHdG levels was observed in very severe COPD (GOLD 4; *p* = 0.781). Procalcitonin concentrations were significantly higher in never smokers than in ever smokers among patients with GOLD 3 (*p* = 0.007) and GOLD 4 disease (*p* = 0.030). No significant differences were identified for the remaining biomarkers.

## 4. Discussion

### 4.1. Serum 8-OHdG and Airflow Limitation Severity

The present study provides additional evidence supporting the clinical relevance of serum 8-OHdG in AECOPD. To our knowledge, this is the first study to quantitatively evaluate serum 8-OHdG levels across GOLD stages in a Caucasian cohort of patients hospitalized for acute exacerbations. The balanced distribution of age, sex, area of residence, smoking status, diabetes, and cardiovascular comorbidities across GOLD categories minimized the potential influence of major confounding factors frequently encountered in COPD populations [3,34,35,36,37].

Serum 8-OHdG concentrations increased progressively with advancing airflow limitation severity and emerged as the only independent predictor of GOLD stage in the ordinal logistic regression model. Furthermore, the observed effect size was moderate, suggesting that the relationship may be clinically meaningful in addition to being statistically significant [8,11].

Our findings are consistent with previous reports linking oxidative DNA damage to COPD severity. El Jundi et al. identified a strong inverse association between serum 8-OHdG levels and pulmonary function parameters, including FEV_1_ and FEV_1_/FVC ratio, in patients with unstable COPD. Similarly, Cao et al. reported that serum 8-OHdG was inversely associated with pulmonary function and positively correlated with inflammatory cytokines and hospitalization-related outcomes. Notably, a twofold increase in serum 8-OHdG concentrations was associated with accelerated annual FEV_1_ decline. Comparable observations have also been reported by Liu et al., who demonstrated increasing plasma 8-OHdG concentrations with worsening GOLD stage and symptom burden during AECOPD. In addition, Yang et al. confirmed a direct relationship between oxidative DNA damage and COPD severity using chromatography-based measurements in peripheral blood mononuclear cells [18,19,20,21].

The biological plausibility of these findings is supported by the central role of oxidative stress in COPD pathophysiology. Chronic exposure to oxidants and persistent airway inflammation promote excessive reactive oxygen species (ROS) production, resulting in oxidative DNA damage and increased formation of 8-OHdG. This process may contribute to epithelial injury, accelerated cellular senescence, impaired tissue repair, and progressive airway remodeling, thereby linking oxidative damage with worsening airflow limitation [1,12,13,45,49,50].

### 4.2. Smoking Status and Oxidative DNA Damage

In addition to its association with GOLD stage, serum 8-OHdG was independently associated with smoking status. Each unit increase in serum 8-OHdG was associated with a 20% increase in the odds of being an ever smoker, highlighting its potential value as a marker of smoking-related oxidative injury [15,18].

These findings are consistent with extensive evidence demonstrating that tobacco smoke is a major source of oxidative stress. El Jundi et al. reported significantly higher serum 8-OHdG concentrations in smokers irrespective of COPD status, whereas Liu et al. observed a stepwise increase in plasma 8-OHdG levels from never smokers to former smokers and current smokers. Similar trends have been documented in pulmonary blood mononuclear cells, lung tissue, and peripheral leukocytes. Furthermore, a systematic review and meta-analysis conducted by Graille et al. identified a robust association between smoking exposure and urinary 8-OHdG concentrations [15,18,20,21,51].

Collectively, these observations support the concept that smoking-related oxidative DNA damage remains detectable at the systemic level and may contribute to the chronic biological burden associated with COPD progression [13,15,18,51].

### 4.3. Differential Biomarker Patterns Across COPD Stages

An important observation of the present study was that the relationship between smoking and serum 8-OHdG differed according to disease severity. Ever smokers exhibited significantly higher serum 8-OHdG concentrations than never smokers in GOLD 1–2 and GOLD 3 disease. However, this difference disappeared in GOLD 4 COPD despite numerically higher values among smokers [18,20].

One possible explanation is the existence of a biological ceiling effect, whereby oxidative stress reaches a plateau in advanced disease. In severe COPD, chronic inflammation, tissue remodeling, and sustained ROS generation may already produce a substantial oxidative burden, reducing the relative contribution of smoking-related oxidative injury. Consequently, differences between smokers and non-smokers may become less apparent as disease severity increases [1,11,12,45,50].

Interestingly, procalcitonin displayed an opposite pattern. Never smokers with GOLD 3 and GOLD 4 disease exhibited significantly higher procalcitonin concentrations than ever smokers. Although the mechanisms underlying this finding remain uncertain, nicotine-mediated immunomodulation may partially contribute to this phenomenon. Alternatively, the observation may reflect differences in exacerbation etiology, with smokers experiencing relatively greater oxidative stress-driven exacerbations and never smokers exhibiting a higher proportion of infection-related events [32,33,52].

Taken together, these findings suggest that oxidative DNA damage and systemic inflammatory responses may follow partially distinct biological pathways during AECOPD. While smoking appears to exert a predominant influence on oxidative DNA damage, infectious triggers may contribute more substantially to procalcitonin responses, particularly in advanced disease [12,32,33].

### 4.4. Clinical Implications and Future Directions

The present findings support the potential role of serum 8-OHdG as a clinically relevant biomarker of oxidative stress in COPD. Compared with conventional inflammatory markers, including leukocyte count, neutrophil percentage, CRP, fibrinogen, and procalcitonin, serum 8-OHdG demonstrated the strongest relationship with both airflow limitation severity and smoking status [8,11].

From a clinical perspective, serum 8-OHdG may provide complementary information regarding the oxidative component of COPD pathophysiology. Such information could contribute to disease characterization and identification of patients with a disproportionately elevated oxidative burden. Moreover, the observed association between smoking status and serum 8-OHdG reinforces the importance of smoking cessation as a strategy to reduce ongoing oxidative injury [1,13,15].

Several questions remain unanswered. The present study was cross-sectional and therefore cannot determine whether elevated 8-OHdG directly contributes to disease progression or simply reflects underlying pathological processes. Prospective longitudinal studies are required to establish whether baseline 8-OHdG concentrations predict future lung function decline, exacerbation frequency, hospitalization, or mortality [12,19].

Beyond COPD, serum 8-OHdG has been investigated as a systemic marker of oxidative DNA damage in several chronic inflammatory diseases, malignancies, and occupational exposure settings, including chronic kidney disease, radiation-exposed workers, diabetes-associated periodontitis, colorectal cancer, and epithelial ovarian carcinoma [53,54,55,56]. These observations support its broader role as an indicator of oxidative stress across diverse pathological conditions. Nevertheless, the present study specifically focused on the clinical relevance of serum 8-OHdG during acute exacerbations of COPD.

Future studies may also investigate whether serum 8-OHdG correlates with imaging biomarkers, including pulmonary ultrasound findings during acute exacerbations, potentially allowing a more comprehensive assessment of oxidative injury and structural lung abnormalities [57]. Other investigations should also evaluate antioxidant defense mechanisms, including glutathione and superoxide dismutase activity, and integrate systemic biomarkers with local airway markers of oxidative stress to provide a more comprehensive assessment of oxidative burden in COPD.

**Figure 1:**
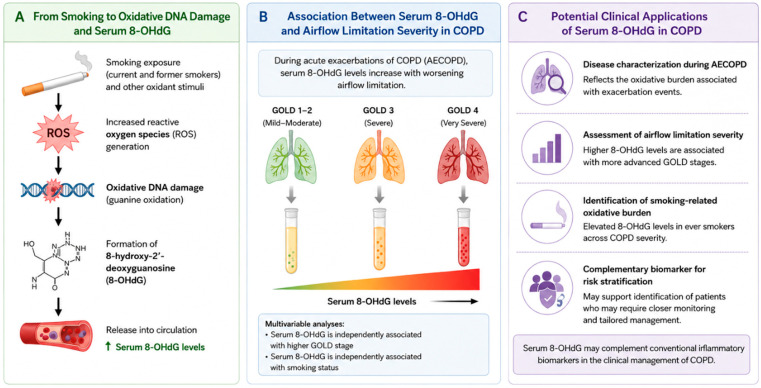
Proposed Relationship between Smoking, Oxidative DNA Damage, and Serum 8-OHdG in COPD.

The relationship between smoking exposure, oxidative DNA damage, serum 8-OHdG levels, and airflow limitation severity in COPD is summarized in Figure 1.

Cigarette smoke and other oxidative stimuli promote reactive oxygen species (ROS) generation, leading to oxidative DNA damage and increased formation of 8-hydroxy-2′-deoxyguanosine (8-OHdG). Higher serum 8-OHdG levels are associated with increasing airflow limitation severity and smoking status during acute exacerbations of COPD (AECOPDs), supporting their potential role as a complementary biomarker for disease characterization and assessment of oxidative burden.

### 4.5. Strengths and Limitations

The strengths of this study include the relatively large cohort of patients hospitalized with AECOPD, the balanced distribution of major clinical confounders across GOLD categories, the inclusion of multiple routinely available inflammatory biomarkers, and the evaluation of serum 8-OHdG across the full spectrum of airflow limitation severity.

Several limitations should also be acknowledged. First, the cross-sectional design precludes causal inference. Second, biomarker measurements were obtained during exacerbation events and were not repeated during recovery, preventing assessment of temporal changes. Third, antioxidant defense markers were not evaluated. Fourth, local airway oxidative stress markers were not measured. Finally, the study was conducted at a single tertiary center, which may limit generalizability to other populations.

## 5. Conclusions

Serum 8-hydroxy-2′-deoxyguanosine (8-OHdG), a marker of oxidative DNA damage, was independently associated with both airflow limitation severity and smoking status in patients hospitalized with acute exacerbations of COPD. Among all biomarkers evaluated, serum 8-OHdG was the only independent predictor of GOLD stage, supporting the relevance of oxidative DNA damage as a distinct biological component of COPD beyond conventional systemic inflammation. These findings highlight the potential clinical value of serum 8-OHdG as a complementary biomarker for disease characterization and assessment of oxidative burden during AECOPD.

## Figures and Tables

**Table 1:** Baseline demographic characteristics and comorbidities across COPD stages.

**COPD Stage**	***n***	**Origin Area**		**Sex**		**Smoking Status**	
		**Urban**	**Rural**	**Male**	**Female**	**Never Smoker**	**Ever Smoker**
GOLD 1–2	48	22 (45.84%)	26 (54.16%)	34 (70.83%)	14 (29.17%)	24 (50.0%)	24 (50.0%)
GOLD 3	82	30 (36.59%)	52 (63.41%)	64 (78.04%)	18 (21.96%)	46 (56.1%)	36 (43.9%)
GOLD 4	46	10 (21.73%)	36 (78.26%)	32 (69.56%)	14 (30.44%)	24 (52.2%)	22 (47.8%)
**COPD stage**	***n***	**Diabetes**		**Cardiovascular condition**			
		**Yes**	**No**	**Yes**	**No**		
GOLD 1–2	48	18 (37.50%)	30 (63.50%)	32 (66.67%)	16 (33.33%)		
GOLD 3	82	35 (47.23%)	47 (52.77%)	56 (68.29%)	26 (31.81%)		
GOLD 4	46	26 (54.17%)	20 (45.83%)	31 (64.58%)	15 (35.42%)		

Data are reported as absolute counts with the corresponding percentages given in parentheses.

**Table 2:** The median values of age and laboratory variables across COPD stages.

Variable	Reference Range	GOLD 1–2	GOLD 3	GOLD 4	*p*	*ε* ^2^
Age (years)		58 (56; 69)	62 (55; 74)	66 (58; 72)	0.480	0.00
8-OHdG (ng/mL)		22.20 (16.50; 36.70)	25.00 (17.40; 37.40)	30.70 (22.40; 42.40)	0.038 *	0.12
ALC (×10^3^/µL)	4.00–10.00	9.71 (7.51; 11.83)	10.07 (8.06; 12.24)	11.60 (7.56; 13.05)	0.809	0.00
NP (%)	45–80	68.95 (60.73; 78.50)	75.50 (64.90; 85.20)	71.90 (66.70; 76.05)	0.409	<0.01
CRP (mg/L)	<5.00	10.37 (4.86; 17.45)	11.04 (6.01; 26.56)	14.04 (4.14; 57.01)	0.649	<0.01
FIB (mg/dL)	200–400	441.00 (377.00; 538.00)	433.00 (323.50; 508.00)	480.50 (374.75; 567.00)	0.240	0.02
PCT (ng/dL)	0.00–0.50	0.06 (0.04; 0.12)	0.06 (0.04; 0.16)	0.05 (0.04; 0.12)	0.839	<0.01

*p*, *p* value; *ε*^2^, epsilon squared; 8-OHdG, 8-hydroxydeoxyguanosine; ALC, absolute leukocyte count; NP, neutrophil percentage; CRP, C-reactive protein; FIB, fibrinogen; PCT, procalcitonin. The second column shows the reference ranges according to Romanian standards. Data in the third, fourth, and fifth columns are presented as medians with their interquartile ranges (25th–75th percentile) given in parentheses. Bold values annotated with asterisks (*) denote significant differences across GOLD stages (Kruskal–Wallis test, *—*p* ≤ 0.05).

**Table 3:** Predictors of airflow limitation severity during AECOPDs.

Variable	OR (95% CI)	*p*	VIF
8-OHdG	2.44 (1.12; 5.31)	0.025 *	1.06
ALC	1.06 (0.94; 1.19)	0.366	1.31
NP	0.98 (0.93; 1.03)	0.483	1.51
CRP	0.99 (0.97; 1.00)	0.130	1.80
FIB	1.00 (0.99; 1.00)	0.414	1.57
PCT	0.94 (0.60; 1.49)	0.797	1.23

8-OHdG, 8-hydroxydeoxyguanosine; ALC, absolute leukocyte count; NP, neutrophil percentage; CRP, C-reactive protein; FIB, fibrinogen; PCT, procalcitonin; OR (95% CI), odds ratio with 95% confidence interval; *p*, *p* value; β, coefficient beta; SE, standard error; Wald (Z), Z-value from the Wald test; VIF, variance inflation factor. Results are reported as odds ratios with the corresponding 95% confidence intervals given in parentheses (second column). Bold values annotated with asterisks (*) in the third column denote significant predictors of airflow limitation severity (Ordinal logistic regression, *—*p* ≤ 0.05).

**Table 4:** Predictors of smoking status during AECOPDs.

Variable	OR (95% CI)	*p*	β	SE	Wald (Z)	VIF
8-OHdG	1.20 (1.05; 1.37)	0.022 *	0.182	0.079	2.29	1.02
ALC	1.06 (0.94; 1.19)	0.366	0.055	0.061	0.90	1.24
NP	0.98 (0.93; 1.03)	0.483	−0.018	0.026	−0.70	1.40
CRP	0.99 (0.97; 1.00)	0.130	−0.014	0.009	−1.51	1.96
FIB	1.00 (0.99; 1.00)	0.414	−0.002	0.003	−0.82	1.72
PCT	0.94 (0.60; 1.49)	0.797	−0.059	0.233	−0.26	1.24

8-OHdG, 8-hydroxydeoxyguanosine; ALC, absolute leukocyte count; NP, neutrophil percentage; CRP, C-reactive protein; FIB, fibrinogen; PCT, procalcitonin; OR (95% CI), odds ratio with 95% confidence interval; β, coefficient beta; SE, standard error; Wald (Z), Z-value from the Wald test; VIF, variance inflation factor. Results are reported as odds ratios with the corresponding 95% confidence intervals given in parentheses (second column). Bold values annotated with asterisks (*) indicate significant predictors (Binary logistic regression, *—*p* ≤ 0.05).

**Table 5:** Biomarker levels according to smoking status within each GOLD stage.

Variable	GOLD 1–2 Never Smoker	GOLD 1–2 Ever Smoker	*p*	GOLD 3 Never Smoker	GOLD 3 Ever Smoker	*p*	GOLD 4 Never Smoker	GOLD 4 Ever Smoker	*p*
8-OHdG (ng/mL)	19.20 (14.65–24.35)	38.30 (20.62–47.77)	**0.045**	18.55 (11.35–25.70)	27.05 (20.48–40.48)	**0.042**	29.95 (22.50–35.25)	38.30 (21.90–45.45)	0.781
ALC (×10^3^/µL)	8.31 (7.13–10.58)	9.94 (8.42–16.14)	0.309	9.95 (7.51–12.94)	9.87 (8.28–11.31)	0.984	10.61 (8.92–12.96)	11.60 (6.67–13.05)	0.926
NP (%)	69.90 (62.00–77.20)	65.35 (60.73–78.62)	0.609	77.80 (65.35–86.75)	70.45 (63.55–75.60)	0.090	69.80 (60.40–74.78)	72.90 (71.30–76.05)	0.340
CRP (mg/L)	10.00 (2.67–23.42)	13.09 (9.10–17.45)	0.372	11.29 (5.92–36.48)	10.79 (5.26–16.86)	0.579	15.01 (6.37–61.45)	28.05 (2.04–53.06)	0.689
FIB (mg/dL)	461.00 (397.50–523.50)	525.00 (383.00–578.25)	0.447	454.50 (370.75–550.75)	412.50 (359.00–491.50)	0.358	452.00 (340.25–509.75)	383.00 (323.50–474.50)	0.441
PCT (ng/mL)	0.06 (0.04–0.11)	0.06 (0.04–0.13)	0.873	0.10 (0.06–0.50)	0.05 (0.04–0.06)	**0.007**	0.10 (0.05–0.14)	0.04 (0.03–0.06)	**0.030**

Abbreviations: 8-OHdG, 8-hydroxy-2′-deoxyguanosine; ALC, absolute leukocyte count; NP, neutrophil percentage; CRP, C-reactive protein; FIB, fibrinogen; PCT, procalcitonin. Data are presented as median (interquartile range). Significant *p* values are shown in bold.

## Data Availability

All the data generated or analyzed during this study are included in this published article.

## Abbreviations

The following abbreviations are used in this manuscript:

AECOPD	Acute Exacerbation of Chronic Obstructive Pulmonary Disease
ALC	Absolute Leukocyte Count
CRP	C-Reactive Protein
COPD	Chronic Obstructive Pulmonary Disease
DNA	Deoxyribonucleic Acid
ELISA	Enzyme-Linked Immunosorbent Assay
FEV_1_	Forced Expiratory Volume in One Second
FIB	Fibrinogen
FVC	Forced Vital Capacity
GOLD	Global Initiative for Chronic Obstructive Lung Disease
HbA1c	Glycated Hemoglobin
NP	Neutrophil Percentage
OR	Odds Ratio
PCT	Procalcitonin
ROS	Reactive Oxygen Species
SE	Standard Error
VIF	Variance Inflation Factor
8-OHdG	8-Hydroxy-2′-Deoxyguanosine
